# The efficacy and safety of panax quinquefolius saponin for heart failure: a systematic review and meta-analysis

**DOI:** 10.3389/fphar.2025.1463609

**Published:** 2025-02-28

**Authors:** Jing Wang, Tianying Chang, Zheng Liang, Yingzi Cui, Xiaodan Wang, Lisha Wang, Hongguang Jin

**Affiliations:** ^1^ School of Traditional Chinese Medicine, Changchun University of Chinese Medicine, Changchun, China; ^2^ EBM office, The Affiliated Hospital to Changchun University of Chinese Medicine, Changchun, China; ^3^ Department of Cardiology, The Affiliated Hospital to Changchun University of Chinese Medicine, Changchun, China

**Keywords:** panax quinquefolius saponin, heart failure, systematic review, meta-analysis, efficacy and safety

## Abstract

**Background:**

Heart failure (HF) is a global health concern, affecting millions of individuals worldwide and leading to significant morbidity and mortality. Despite advances in conventional therapeutic strategies, the prognosis for HF patients remains challenging, and there is a constant search for novel therapeutic options. Among these, Panax quinquefolius saponin (PQS) has demonstrated promising pharmacological properties that may benefit HF. However, the efficacy and safety of PQS for HF have not been comprehensively evaluated.

**Objective:**

This systematic review and meta-analysis aim to provide a more reliable estimation of the efficacy and safety of PQS for HF. This will help clinicians make informed decisions regarding the potential use of PQS in managing HF patients.

**Methods:**

We comprehensively and systematically searched for published randomized controlled trials (RCTs) in the following eight electronic databases: PubMed, Cochrane Library, EMBASE, Web of Science (WOS), China National Knowledge Infrastructure (CNKI), China Science and Technology Journal Database (VIP), Wanfang Data, and China Biology Medicine Database (CBM) from database inception to March 2024. The Cochrane risk of bias (ROB 2.0) assessment tool was used for quality assessment, and Review Manager (RevMan, version 5.4) was used for meta-analysis. Mean difference (MD), 95% credible interval (CI), and relative risk (RR) estimates were calculated under a random-effects model. We also used GRADE profiler (GRADEpro, version 3.6) to analyze the quality of outcomes. In addition, the protocol has been registered in International Platform of Registered Systematic Review and Meta-analysis Protocols (INPLASY) under registry number 202440050.

**Results:**

This study included nine RCTs involving a total of 952 patients with HF. The results of a meta-analysis under a random-effects model showed that adjuvant PQS therapy significantly increased LVEF (MD = 6.23, 95% CI [4.35, 8.12], P < 0.00001), 6MWTD (MD = 25.26, 95% CI [8.23, 42.30], P = 0.004), and decreased BNP/NT-pro-BNP (MD = −187.94, 95% CI [−267.20, −108.67], P < 0.00001), LVEDV (MD = −22.83, 95% CI [−42.79, −2.87], P = 0.02), LVEDD (MD = −4.76, 95% CI [−5.77, −3.74], P < 0.00001), and LVESV (MD = −11.86, 95% CI [−19.89, −3.83], P = 0.004) in patients with HF.

**Conclusion:**

The evidence provided by this systematic review suggests that adjunctive PQS therapy for HF can improved clinical efficacy and holds potential advantages in improving cardiac function and increasing exercise tolerance. However, given the limitations inherent in this review, the conclusions of this study should be interpreted cautiously. Therefore, in clinical practice, it is recommended that physicians tailor treatment strategies according to the specific circumstances of individual patients.

**Systematic Review registration:**

https://inplasy.com/?s=202440050

## 1 Introduction

Currently, the disability and mortality rates of heart disease are extremely high (GBD Causes of Death Collaborators, 2021). Among them, heart failure (HF) is the end stage of many cardiovascular diseases. At the same time, HF is a global health concern, affecting over 64 million people worldwide and leading to significant morbidity and mortality (Savarese et al., 2023). It is a clinical syndrome characterized by symptoms and/or signs resulting from structural and/or functional cardiac abnormalities, supported by elevated natriuretic peptide levels and/or objective evidence of pulmonary or systemic congestion (Bozkurt et al., 2021). HF presents clinical symptoms that include dyspnea, orthopnea, paroxysmal nocturnal dyspnea, and peripheral edema. The majority of the affected population is elderly, and the incidence of the disease increases with age (McDonagh et al., 2021). However, there has been a growing trend towards occurring in younger individuals in recent years (McDonagh et al., 2022). The Report on Cardiovascular Health and Diseases in China 2023: an Updated Summary stated that there are 8.9 million people with HF, which the total cost of HF hospitalization in 2022 totaled 17.06 billion yuan (Diseases et al., 2024). Furthermore, the American Heart Association forecasts that the total cost of treating HF patients will increase from $31 billion in 2012 to $70 billion in 2030 (Heidenreich et al., 2013). HF leads to a marked decline in the quality of life for most patients and imposes a heavy economic burden on individuals and society (GBD Disease and Injury Incidence and Prevalence Collaborators, 2017, McDonagh et al., 2023), making it a significant public health problem.

The interaction of multiple etiological and pathophysiological mechanisms constitutes the complex pathophysiology of HF (Chen et al., 2023). HF is primarily myocardial injury, leading to a progressive decline in the structure and function of the heart, culminating in end-stage heart disease (Lin et al., 2021). HF is mainly caused by risk factors for cardiovascular disease, including coronary heart disease (CHD), hypertension, atrial fibrillation, and diabetes (Ziaeian and Fonarow, 2016). In addition to cardiovascular diseases, non-cardiovascular diseases can also lead to HF. It is currently understood that HF is a chronic, progressive condition. The pathogenesis of HF involves multiple aspects (Maruyama and Imanaka-Yoshida, 2022; Aimo et al., 2020; Fontes et al., 2020; Abernethy et al., 2018). Firstly, activation of the neuroendocrine system leading to myocardial remodeling is a key factor in the onset and progression of HF (Hartupee and Mann, 2017). This includes adrenaline, noradrenaline, and the renin-angiotensin-aldosterone system. The excessive activation of these systems can result in myocardial remodeling, including cardiomyocyte hypertrophy and interstitial fibrosis, ultimately leading to impaired cardiac function. Secondly, myocardial metabolism is also a critical aspect of HF pathogenesis. Cardiomyocytes require significant energy to maintain normal contractile and diastolic functions, and any abnormalities affecting myocardial energy metabolism may lead to myocardial dysfunction (Da Dalt et al., 2023). Chronic inflammatory reactions can promote myocardial remodeling, cell apoptosis, and interstitial fibrosis, thereby causing a decline in myocardial function. Additionally, adipokines and cytokines are involved in regulating the pathogenesis of HF. For example, adipokines and cytokines associated with obesity may affect cardiac function through various pathways, including inducing cardiomyocyte hypertrophy, inflammatory reactions, and fibrosis (Packer, 2018). Damage to endothelial cells leading to insufficient nitric oxide secretion can result in impaired nitric oxide-soluble guanylate cyclase-cyclic guanosine monophosphate (NO-sGC-cGMP) signaling pathway, which also has significant effects on myocardial fibrosis and remodeling (Paulus and Tschöpe, 2013). Recent study has further highlighted the role of inflammatory cytokines, such as interleukin-6 (IL-6) and tumor necrosis factor-alpha (TNF-α), in promoting myocardial inflammation and adverse remodeling in HF (Murphy et al., 2020). Mitochondrial dysfunction is also an essential factor in the occurrence and development of HF (Hinton et al., 2024; Zhou et al., 2020). It is a complex disease, and its pathogenesis involves the interaction of multiple factors. A thorough understanding of these mechanisms is crucial for preventing and treating HF. Based on the current pathogenesis, the conventional foundational pharmacotherapy primarily includes diuretics, renin-angiotensin system inhibitors (RASI), β-blockers, mineralocorticoid receptor antagonist (MRA), sodium-glucose co-transporter two inhibitors (SGLT2i), and soluble guanylate cyclase stimulators (SGC) (Chinese Society of Cardiology, 2024), etc. These medications can reduce myocardial oxygen consumption, improve clinical symptoms and quality of life, and enhance exercise tolerance, thereby preventing or reversing ventricular remodeling, and reducing HF readmissions. However, these medications still cannot fully address the fundamental issue of HF (Wilcox et al., 2020), as mortality and readmission rates remain high. Therefore, there is a continual need to create new anti-HF medications to prevent and treat HF effectively.

Panax quinquefolius (PQ), namely American ginseng, commonly known as North American ginseng, has been recognized as an herb of the genus Panax in the United States and Canada, and its roots and rhizomes have been used widely for more than 300 years in China (Xing et al., 2019; Ma et al., 2017). Panax quinquefolius saponin (PQS) is the main active component extracted from the stems and leaves of PQ and can be applied in the clinic of various diseases (Wang et al., 2015a). The saponins in the roots, stems, and leaves of PQ mainly include ginsenosides Rb1, Rb2, Rg1, Rb3, Rc, Rd, and Re (Xing et al., 2019). However, the content of ginsenosides in stems and leaves was significantly higher than in roots. PQ stems and leaves can be harvested from September to October every year, and ginsenosides produced from stems and leaves have the characteristics of lower cost (Zhang et al., 2022). Ginsenoside Rb3 (GRb3) is one of the main components absorbed into the bloodstream from PQS. Previous studies have shown its estrogen receptor agonistic effects, making it one of the pharmacological bases for PQS to exert estrogen-like cardiovascular protective effects (Pan et al., 2014; Wang et al., 2022). Additionally, a study has shown that ginsenoside Rg1 (GRg1) can influence factor secretion, manifested by reducing the secretion of collagen and TGF-β under high glucose conditions, thereby inhibiting the proliferation of cardiac fibroblasts. PQS can also inhibit the signaling of R-Smads/Co-Smad through the TGF-β1/Smads pathway, suppress the expression of connective tissue growth factor, thereby affecting myocardial fibrosis, and inhibiting HF (Zhang et al., 2023). Several previous pharmacological studies have found that PQS has the effects of being anti-oxidant, improving immunity and ventricular remodeling, anti-fatigue, increasing energy storage in ischemic myocardium, anti-inflammation, free radical scavenging, kidney protection, improving myocardial energy metabolism (Wang et al., 2015b; Tang et al., 2016; Kim et al., 2019; Liu et al., 2015; Zhang et al., 2019). A present study demonstrates that North American ginseng attenuates β-adrenergic-activation-induced cardiac hypertrophy as well as HF by preventing PKA activation and CREB phosphorylation (Tang et al., 2016). Currently, a main patent preparation containing PQS components approved for market by the State Food and Drug Administration is Xinyue Capsule (launched in 2003, commercial name as Xinyue capsule, Z20030073) (Wang et al., 2023). It has been widely used for treating CHD for over 20 years. The Chinese Expert Consensus on the Clinical Application of Xinyue Capsule recommends the concurrent use of Xinyue Capsule with conventional medicine treatments for chronic heart failure (CHF). After treatment, it significantly improves left ventricular ejection fraction (LVEF) in patients, clinical heart function grading, and lowers B-type natriuretic peptide (BNP) levels (Liu et al., 2014). Prior basic research has confirmed that PQS combined with dual antiplatelet therapy can improve ventricular remodeling in rats with acute myocardial infarction (Kou et al., 2018). Additionally, randomized controlled trials (RCTs) reports have shown that the application of PQS formulations on top of conventional medicine treatment can improve clinical symptoms, enhance cardiac function, and increase LVEF in patients with HF. This significantly improves the quality of life for patients. However, current RCTs suffer from limitations such as small sample sizes and low quality. Furthermore, the lack of comprehensive and objective systematic reviews and meta-analyses also leads to a lack of clear understanding of the overall efficacy and safety of PQS for HF. Therefore, this systematic review and meta-analysis aims to evaluate the efficacy and safety of PQS for HF, using high-quality clinical RCTs. The objective is to provide reliable evidence-based medicine for clinical practice.

## 2 Methods

The systematic review and meta-analysis were reported according to the Preferred Reporting Item for Systematic Reviews and Meta-Analyses (PRISMA) guidelines (Page et al., 2021). No additional ethical approval or written consent from patients is required, as all data can be freely obtained from public databases. This protocol has been registered in International Platform of Registered Systematic Review and Meta-analysis Protocols (INPLASY), and the registration number is 202,440,050.

### 2.1 Search strategies

We systematically searched for published RCTs in the following eight electronic databases: PubMed, Cochrane Library, EMBASE, Web of Science (WOS), China National Knowledge Infrastructure (CNKI), China Science and Technology Journal Database (VIP), Wanfang Data and China Biology Medicine Database (CBM) from database inception to March 2024 with no language restriction. The Medical Subject Headings (MeSH) and keywords used for the search included: “heart failure,” “Cardiac Failure,” “Congestive Heart Failure,” “Heart Failure, Right-Sided,” “Heart Failure, Left Sided,” “heart incompetence,” “cardiac backward failure,” “chronic heart insufficiency,” “Heart Decompensation,” “Myocardial Failure,” “Panax quinquefolius saponin,” “PQS,” and “Xinyue capsule.” Please refer to the Supplementary Materials for details on the search strategy.

### 2.2 Study selection and eligibility criteria

#### 2.2.1 Study design

This systematic review only included RCTs that meet the eligibility criteria for inclusion. The following were the specific inclusion criteria.

#### 2.2.2 Patients

Participants who have been definitively diagnosed with HF.

#### 2.2.3 Interventions

HF patients meet the indications for PQS treatment. PQS preparations alone or combined with PQS preparations based on conventional treatment.

#### 2.2.4 Control group interventions

The control group received conventional drug therapy or a placebo. Conventional drugs include diuretics, RASI, MRA,β-blockers, cardiotonic, and vasodilators.

#### 2.2.5 Outcomes

Referring to the Clinical Practice Guidelines for the Management of Heart Failure with Chinese Patent Medicine (2021) (Diseases, 2022), and consulting clinical doctors, the following outcome measures were ultimately determined: (1). Primary outcomes included LVEF, BNP or N-terminal pro-BNP (NT-pro-BNP), and 6-minute walk test distance (6MWTD). (2). Secondary outcomes included left ventricular end-diastolic volume (LVEDV), left ventricular end-diastolic diameter (LVEDD), left ventricular end-systolic volume (LVESV), and adverse events.

#### 2.2.6 Exclusion criteria

The exclusion criteria used in this review are as follows: (1). The study conducted on non-human subjects; (2). Literature available only in abstract form without full-text access; (3). The study has insufficient or irrelevant data that cannot be used for meta-analysis or to draw meaningful conclusions. (4). The same clinical data was published by the same author in different journals.

### 2.3 Data extraction

We used EndNoteX9 to manage the retrieved records from the eight electronic databases mentioned above. Two researchers (Jing Wang and Zheng Liang) independently extracted data from the included studies strictly according to eligibility criteria and cross-checked the extracted data. If there is any disagreement about the extracted data, it can be discussed with a third researcher (Tianying Chang). The details of the extracted information were as follows: basic characteristics of studies (author, year, title, study location, study design, sample size, New York Heart Association (NYHA) cardiac function classification); interventions (dosing regimen, dose, treatment duration, follow-up time); subject characteristics (criteria for diagnosis of the disease, age, sex, course of disease); and primary outcomes (LVEF, BNP/NT-pro-BNP, 6MWTD), secondary outcomes (LVEDV, LVEDD, LVESV and adverse events).

### 2.4 Assessment of risk of bias

We utilized the Risk of Bias assessment tool recommended by the Cochrane Handbook (ROB 2.0) to evaluate the risk of bias in the included studies. Two reviewers (Jing Wang and Tianying Chang) independently conducted the assessment based on the ROB criteria. ROB 2.0 assesses bias across five domains: randomization process, deviations from intended interventions, missing outcome data, outcome measurement, and selective outcome reporting. Each study can be assessed as having high RoB, some concerns, or low RoB. In discrepancies, consensus can be reached through discussion between the two reviewers or resolved through consultation with a third party (Zheng Liang).

### 2.5 Statistical analysis and synthesis

We used RevMan (version 5.4) by the Cochrane Collaboration to analyze and synthesize the data. We synthesized available outcomes data from more than two studies for meta-analysis. Continuous outcomes data were presented as mean differences (MD) with 95% credible interval (CI), while for dichotomous data, risk ratios (RR) with 95% CI were used for analysis. We applied a random-effects model to conduct meta-analyses for all outcome measures and assessed heterogeneity using the I^2^ test. Sensitivity analysis and subgroup analysis were performed when statistical heterogeneity was present among included studies (I^2^ ≥ 30% and P ≤ 0.10). Subgroup analysis was conducted based on predefined variables to explore potential sources of heterogeneity: intervention measures in the treatment group (sole use of PQS preparations or in combination with other traditional Chinese medicines), intervention measures in the control group (selection of conventional therapeutic drugs), and duration of treatment (T ≤ 12 weeks, T > 12 weeks). Sensitivity analysis was performed using the leave-one-out method to ensure the robustness of the results. We sequentially excluded each study from the meta-analysis to evaluate its impact on the overall effect size and heterogeneity.

To assess the certainty of evidence. We used the Grading of Recommendations Assessment, Development and Evaluation (GRADE) to grade the quality of evidence. We considered five aspects to determine whether to downgrade the evidence level: risk of bias (moderate risk downgrades by one level, high risk downgrades by two levels), inconsistency, indirectness, imprecision, and publication bias. Based on these assessments, the quality of evidence was categorized into four levels: high, moderate, low, and very low (Balshem et al., 2011). This work was independently conducted by two authors (Wang Jing and Tianying Chang) using GRADE profiler (GRADEpro, version3.6). If disagreements arise during the process, resolution could be achieved through discussion or consultation with a third author (Hongguang Jin).

## 3 Results

### 3.1 Literature screening results

**FIGURE 1:**
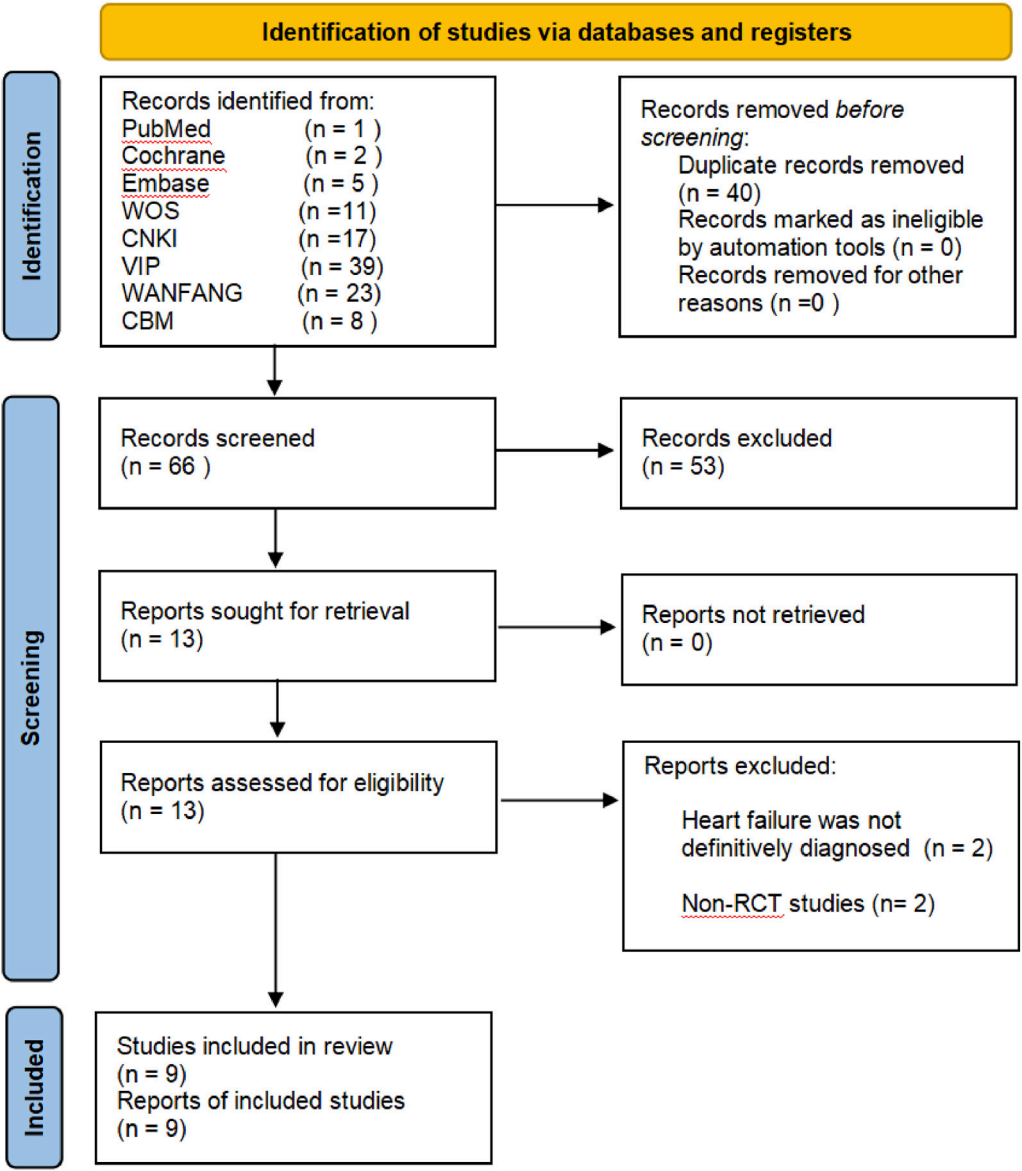
Study screening flow chart.

A total of 106 records were identified through a preliminary search in eight electronic databases. Then, we applied EndNoteX9 document management software to remove 40 duplicate records, and 66 records remained. After we had read the titles and abstracts, 53 papers were excluded for the following different reasons: ineligible studies (n = 48), conference papers (n = 2), dissertations (n = 2), and animal experiments (n = 1). The full-text of the 13 RCTs was further reviewed, of which 9 studies met the inclusion criteria, unclear diagnosis (n = 2), and non-RCTs (n = 2) were excluded. In the end, the meta-analysis included nine RCTs (Xie et al., 2023; Guan et al., 2023; Wang et al., 2021; Qian et al., 2021; Wang, 2018; Shi, 2014; Yu, 2013; Guo, 2012; Han et al., 2011). The study screening flow chart is shown in Figure 1.

### 3.2 Characteristics of included studies

The included nine RCTs, with a total of 952 patients with HF (480 in the PQS intervention group and 472 in the control group, comprising 398 females and 554 males). The ages of the patients ranged from 42 to 83 years old. The duration of treatment ranged from 16 days to 24 weeks. All of these RCTs were conducted in China and published in Chinese between 2011 and 2023. All intervention groups in the studies were administered PQS preparations (Xinyue Capsule) combined with routine anti-HF drug treatments (Xie et al., 2023; Guan et al., 2023; Wang et al., 2021; Qian et al., 2021; Wang, 2018; Shi, 2014; Yu, 2013; Guo, 2012; Han et al., 2011). In these RCTs, five studies (Xie et al., 2023; Wang et al., 2021; Qian et al., 2021; Shi, 2014; Han et al., 2011) reported the classification of NYHA heart function (NYHA classⅡ: 176 patients; NYHA class Ⅲ: 268 patients; NYHA class Ⅳ: 116 patients), and four studies (Qian et al., 2021; Yu, 2013; Guo, 2012; Han et al., 2011) mentioned that the course of HF ranged from four to 9 years. Additionally, one study (Xie et al., 2023) investigated specifically adverse events (intervention group: Two patients of dry cough, three patients of gastrointestinal reactions, two patients of hypokalemia; one patient of low blood pressure; control group: three patients of dry cough; one patient of gastrointestinal reactions, one patient of hypokalemia; four patients of low blood pressure), three studies (Qian et al., 2021; Guo, 2012; Han et al., 2011) had no adverse reactions during the course of treatment. The specific characteristics are shown in Table 1.

**TABLE 1:** Characteristics of included studies.

Study	Sample size		Age		Male/Female		Classification of NYHA heart function (Ⅱ/Ⅲ/Ⅳ)		Course of disease (years)		Intervention(s)		Treatment duration	Outcomes	Adverse events
	T	C	T	C	T	C	T	C	T	C	T	C			
Guan et al. (2023)	40	40	62.35 ± 12.06	61 ± 13.51	25/15	28/12	NA	NA	NA	NA	CGT + QLQXC 1.2 g tid + XYC 0.6 g tid	Spironolactone tablets 20 mg qd + Benalapril hydrochloride tablets 5 mg qd + Metoprolol tartrate tablets 25 mg bid	6 months	LVEF, NT-proBNP, 6MWTD, LVESD, LVEDD	T: Two patients of dry cough, three patients of gastrointestinal reactions, two patients of hypokalemia; one patient of hypotension C: Three patients of dry cough; one patient of gastrointestinal reactions,one patient of hypokalemia; four patients of hypotension
Xie et al. (2023)	75	75	63.52 ± 3.25	63.47 ± 3.24	41/34	39/36	12/46/17	10/45/20	NA	NA	CGT + XYC 0.6 g tid	RT + Sacubitril valsartan sodium tablets	8 weeks	LVEF, NT-proBNP, LVEDV, LVEDD, MLHFQ	Not reported
Wang et al. (2021)	80	80	68.47 ± 2.25	69.02 ± 2.10	45/35	47/33	32/27/21	34/28/18	NA	NA	RT + XYC 0.6 g tid	RT (such as: Benazepril, Furosemide, Spironolactone, Isosorbide mononitrate, digoxin, etc.)	4 weeks	LVEF, BNP, LVEDD, SV, CO, ADH, SF-36	Not reported
Qian et al. (2021)	43	43	63.7 ± 17.5	65.5 ± 17.9	28/15	26/17	14/27/2	16/23/4	7.5 ± 1.6	7.9 ± 1.8	RT + XYC 0.6 g tid	RT	24 weeks	LVEF, BNP, 6MWTD	No occurred
Wang (2018)	65	71	63.5 ± 10.9	61.3 ± 9.5	39/26	44/27	NA	NA	NA	NA	RT + XYC 0.6 g tid	RT	24 weeks	LVEF, BNP	Not reported
Shi (2014)	40	40	66.5 ± 10.2	63.8 ± 9.1	22/18	24/16	16/13/11	17/10/13	NA	NA	RT + XYC 0.6 g tid	RT	3 months	LVEF	Not reported
Yu (2013)	38	26	52 ± 3.1	51 ± 2.4	21/17	15/11	NA	NA	6.5 ± 2.3	6.4 ± 1.2	RT + XYC 0.6 g tid	RT	4 weeks	NA	Not reported
Guo (2012)	45	45	57.1	56.3	23/22	25/20	NA	NA	4.3	4.1	CGT + XYC 0.6 g tid	Digoxin 0.25 mg qd + Hydrochlorothiazide 50 mg qd + Betalok 6.25 mg bid + Spironolactone 20 mg qd	19.5 ± 2.7 days	LVEF, LVEDV, LVESV	No occurred
Han et al. (2011)	54	52	61.7 ± 16.2	61.2 ± 18.4	33/21	29/23	12/25/5	13/24/5	6.6 ± 1.5	6.2 ± 1.5	RT + XYC 0.6 g tid	RT	4 weeks	LVEF, LVEDV, LVESV, 6MWTD	No occurred

T, intervention group; C, control group; CGT, Control group treatment; QLQXC, Qiliqiangxin Capsule; XYC, Xinyue Capsule; RT, Routine Treatment; qd, once daily; bid, twice daily; tid, three times daily; NA, Data Missing; LVEF, left ventricular ejection fraction; BNP, B-type natriuretic peptide; NT-proBNP, N-terminal pro-BNP; 6MWTD, 6-min walk test distance; LVEDV, left ventricular end-diastolic volume; LVEDD, left ventricular end-diastolic diamete; LVESD, left ventricular end-systolic diamete; LVESV, left ventricular end-systolic volume; SV, stroke volume; CO, Cardiac Output; ADH, antidiuretic hormone; SF-36, the MOS item short from health survey; MLHFQ, Minnesota Living with Heart Failure Questionnaire.

### 3.3 Assessment of risk of bias

**FIGURE 2:**
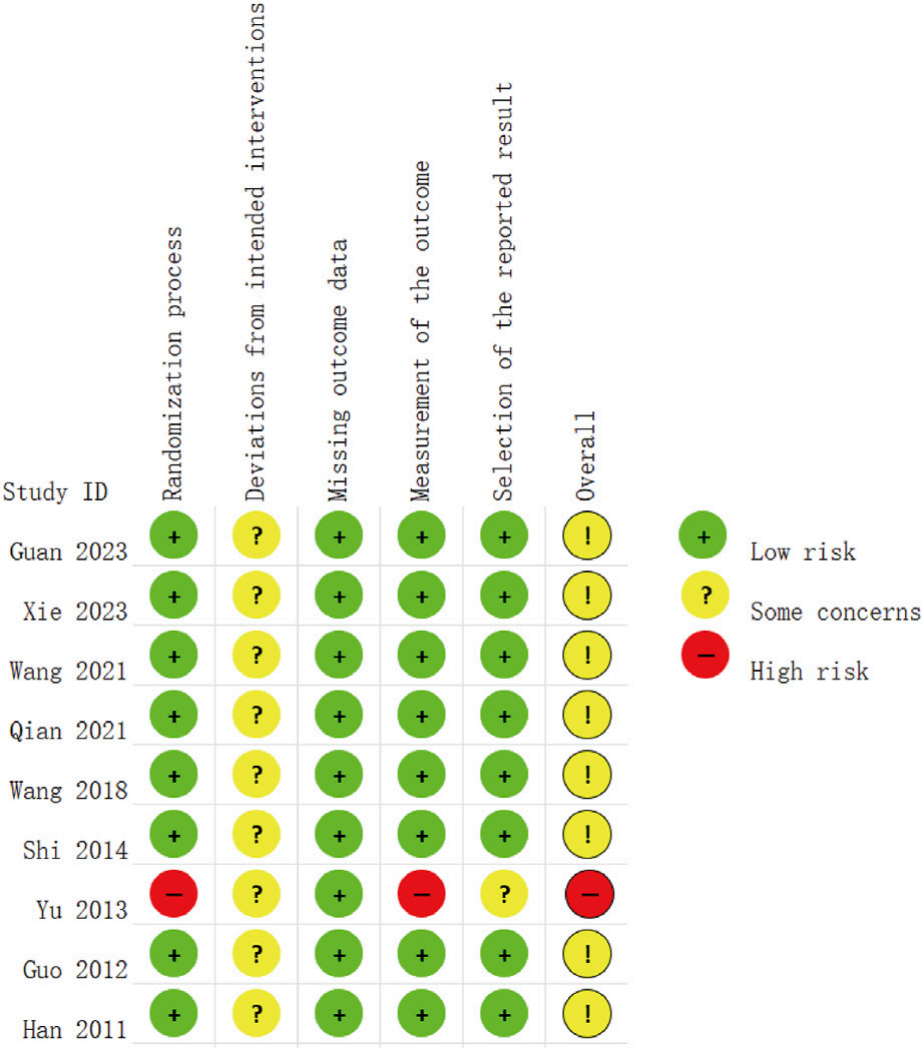
Risk of bias summary.

**FIGURE 3:**
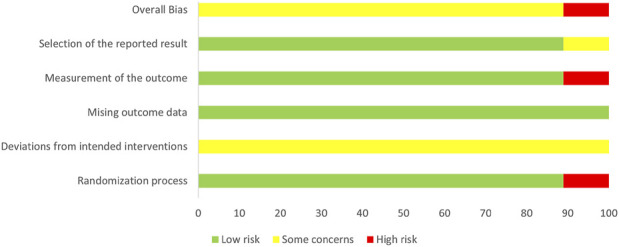
Risk of bias graph.

The risk assessment was independently conducted by two researchers, followed by a double-check. The bias risk of the included studies is presented in Figures 2, 3. Of the nine included RCTs, one study (Yu, 2013) was deemed to have a high RoB, while eight studies (Xie et al., 2023; Guan et al., 2023; Wang et al., 2021; Qian et al., 2021; Wang, 2018; Shi, 2014; Guo, 2012; Han et al., 2011) raised some concerns regarding the overall RoB assessment. The trial with a high RoB only mentioned randomization without providing detailed information, and it did not utilize clearly defined, objective outcome measures. We found that the third domain (Measurement of the outcome) assessing all nine trials was at low risk. However, there were some concerns identified in the assessment of the second domain (Deviations from intended interventions), primarily due to the lack of detailed mention of the use of blinding in the text. Overall, we consider the risk of bias in the nine studies to be low, as there were no significant differences observed in outcome measurements between groups and all study data were complete.

### 3.4 Efficacy analyses of PQS for heart failure

#### 3.4.1 Primary outcomes

##### 3.4.1.1 Left ventricular ejection fractions (LVEF)

**FIGURE 4:**
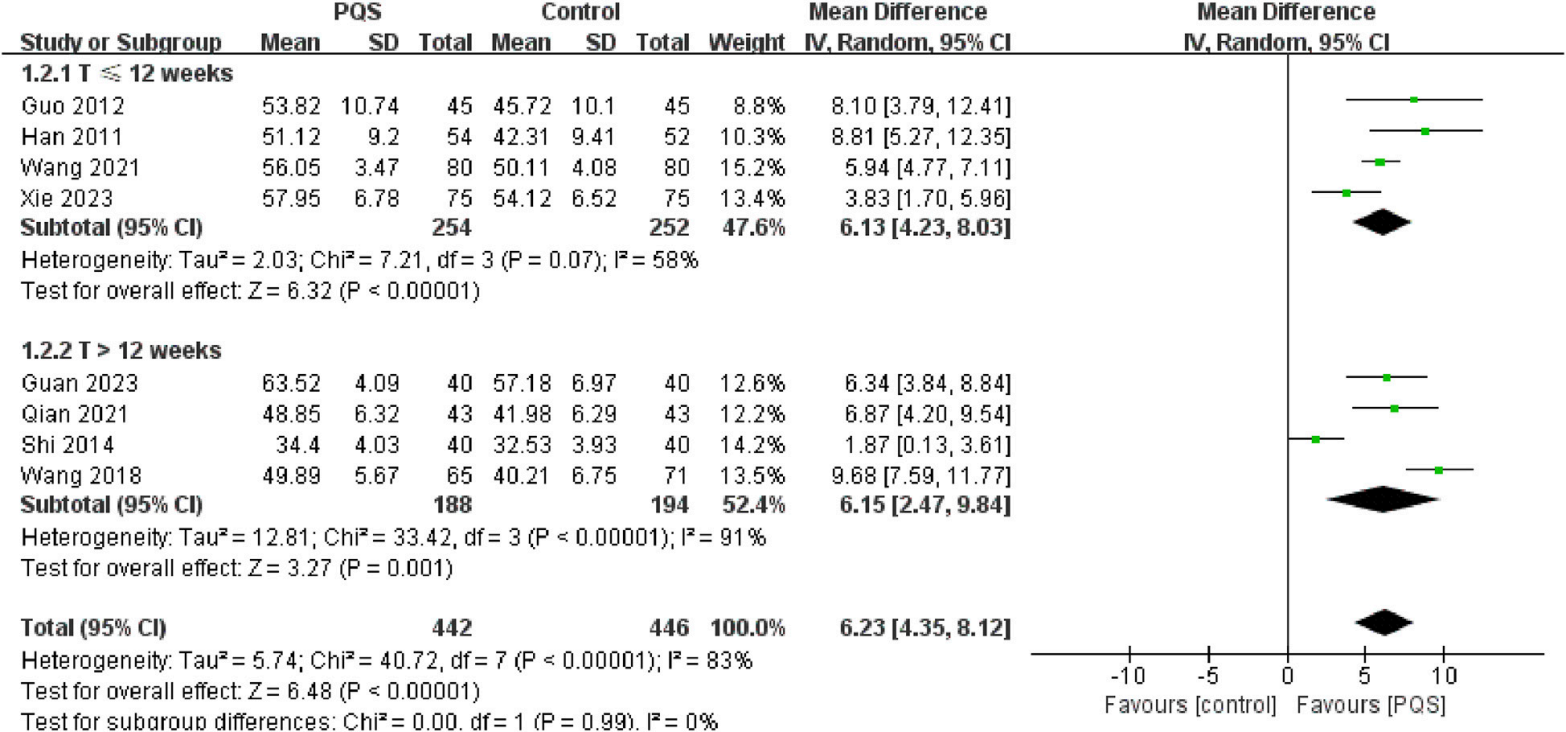
Meta-analysis of the effects of PQS on LVEF.

LVEF is a measure of the systolic function of the heart (Baman and Ahmad, 2020). It was reported by eight studies. Random-effects models were used to analyze pooled data (n = 888, MD = 6.23, 95% CI [4.35, 8.12], P < 0.00001) for significant heterogeneous distribution (P < 0.00001, I^2^ = 83%). Meta-analysis showed that PQS combined with routine anti-HF drug treatments improved LVEF more than routine anti-HF drug treatment. We divided into two groups for subgroup analysis according to different treatment durations (≤12 weeks and >12 weeks). Both subgroups improved LVEF. Meta-analysis under the random-effects model showed that the LVEF was significantly higher than that of the control group (Figure 4).

##### 3.4.1.2 BNP/NT-pro-BNP

**FIGURE 5:**
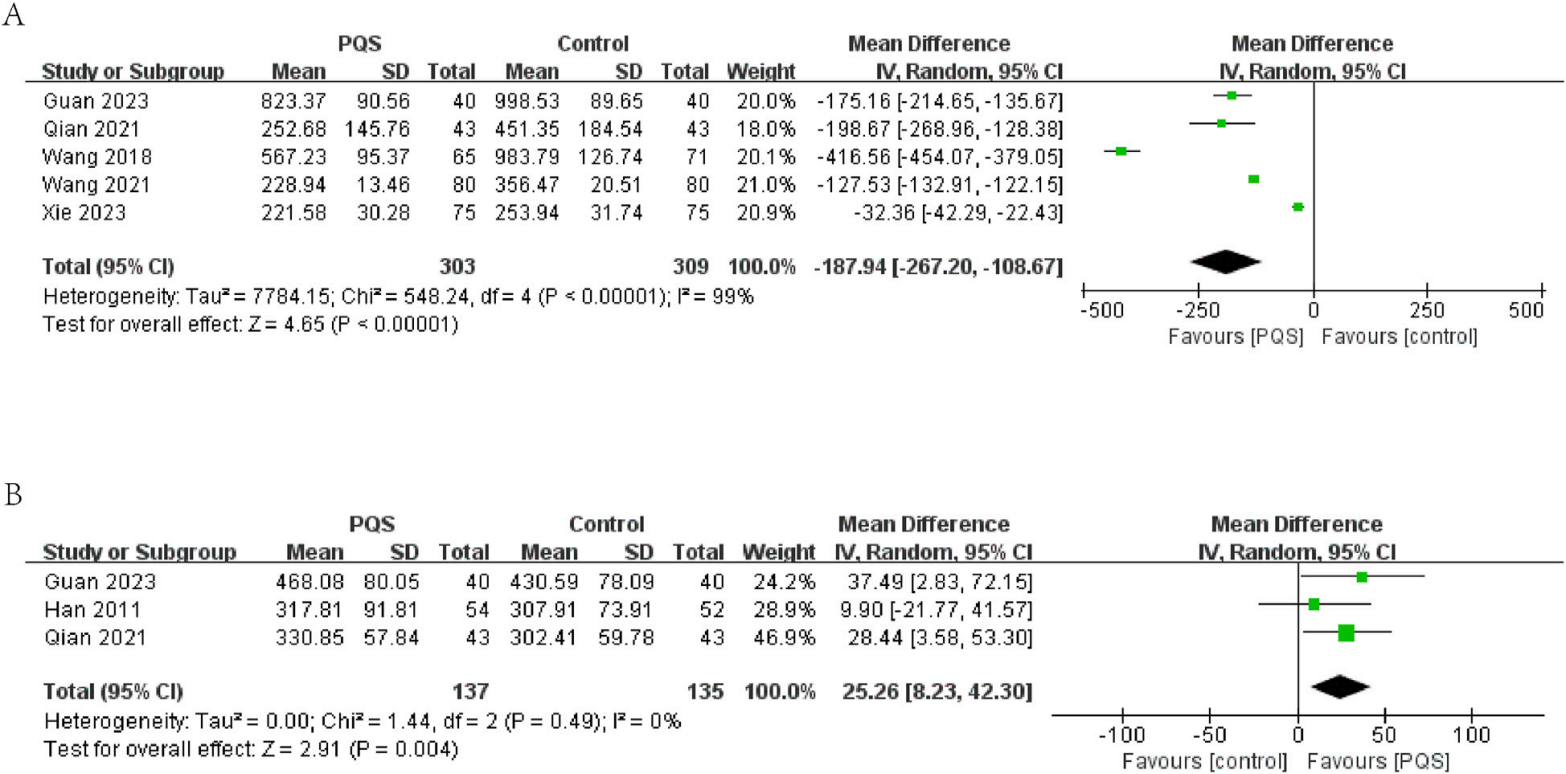
Effects of PQS on **(A)** BNP/NT-pro-BNP, **(B)** 6-min walk test distance (6MWTD).

In five studies, BNP or NT-pro-BNP was evaluated. The results showed significant heterogeneity among these studies (P < 0.00001, I^2^ = 99%). The results of meta-analysis under a random-effects model showed that BNP or NT-pro-BNP was lower in the PQS groups (n = 612, MD = −187.94, 95% CI [−267.20, −108.67], P < 0.00001) (Figure 5A).

##### 3.4.1.3 6-min walk test distance (6MWTD)

6MWTD (measurement unit: meter) was evaluated in three studies. The meta-analysis under a random-effects model showed no significant heterogeneity (P = 0.49, I^2^ = 0%). The intervention group demonstrated a significant increase in 6MWTD compared to the control group (n = 272, MD = 25.26, 95% CI [8.23, 42.30], P = 0.004). This suggests that PQS therapy may improve exercise tolerance in HF patients (Figure 5B).

#### 3.4.2 Secondary outcomes

##### 3.4.2.1 LVEDV

**FIGURE 6:**
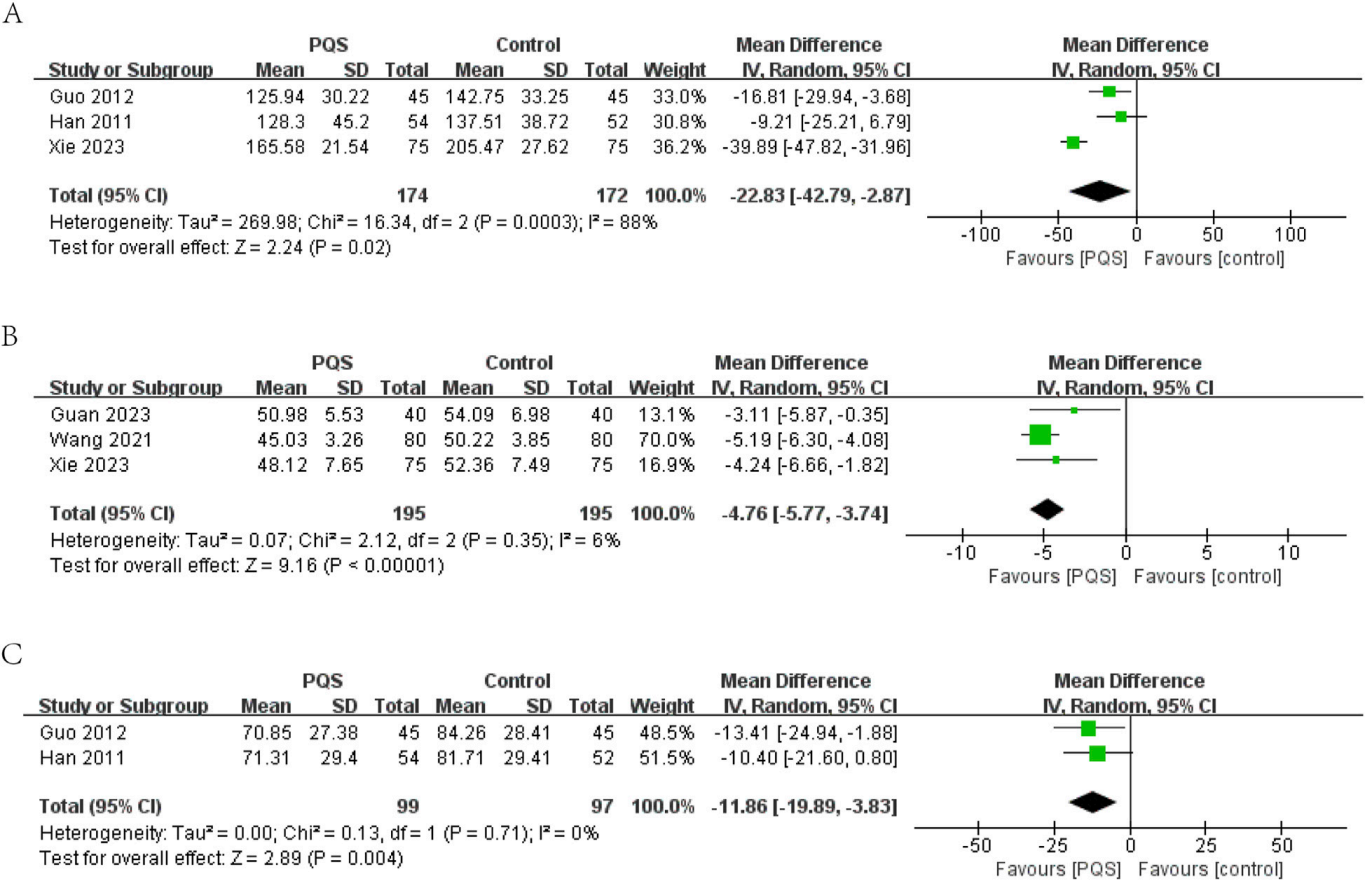
Meta-analysis of the effects of PQS on **(A)** LVEDV, **(B)** LVEDD, **(C)** LVESV.

LVEDV was reported in three studies. Random-effects models were used to analyze data (MD = −22.83, 95% CI [−42.79, −2.87], P = 0.02) for significant heterogeneity (P = 0.0003, I^2^ = 88%). Meta-analysis showed that PQS preparation was more significant in reducing LVEDV (Figure 6A).

##### 3.4.2.2 LVEDD

LVEDD (measurement unit: millimeter) was reported in three studies. The meta-analysis under a random-effects model showed no significant heterogeneity (P = 0.35, I^2^ = 6%). The PQS group exhibited a significant reduction in LVEDD compared to the control group (n = 390, MD = −4.76, 95% CI [−5.77, −3.74], P < 0.00001), indicating improved cardiac remodeling (Figure 6B).

##### 3.4.2.3 LVESV

LVESV (measurement unit: milliliter) was reported in two studies. The meta-analysis under a random-effects model showed no significant heterogeneity (P = 0.71, I^2^ = 0%). The PQS group demonstrated a significant reduction in LVESV compared to the control group (n = 196, MD = −11.86, 95% CI [−19.89, −3.83], P = 0.004), further supporting the beneficial effects of PQS on cardiac function (Figure 6C).

##### 3.4.2.4 Adverse events

Among the nine included studies, only one (Guan et al., 2023) provided detailed information on adverse events, three mentioned that no adverse events occurred, and the remaining studies did not report on adverse events. The observed adverse events included dry cough, gastrointestinal reactions, hypokalemia, and hypotension. A total of eight patients occurred adverse events in the intervention group (two patients with dry cough, three patients with gastrointestinal reactions, two patients of hypokalemia; one patient with hypotension), and their overall incidence of adverse events was 20%, but a total of nine patients occurred in the control group (three patients of dry cough; one patient of gastrointestinal reactions, one patient of hypokalemia; four patients of low blood pressure), for an overall adverse event rate of 22.5%. However, the study did not report the severity of adverse events in detail. The adverse events are in detail in Table 1.

### 3.5 Sensitivity analysis

In our meta-analysis, significant heterogeneity was found in the comparisons of LVEF, BNP/NT-proBNP, and LVEDV. However, due to the limited number of included studies, we only conducted a series of sensitivity tests on those with more than five studies to ensure the robustness of our findings. The leave-one-out analysis showed that the heterogeneity of BNP/NT-pro-BNP remained unchanged regardless of which study was excluded (P < 0.00001, I^2^ = 99%) and the direction of the overall effect for BNP/NT-pro-BNP remained consistent, indicating that no single study had a disproportionate influence on the results. For LVEF, excluding one study (Shi, 2014) reduced heterogeneity (I^2^ from 83% to 67%) but did not significantly alter the overall effect size. Thus, the results of this analysis strengthen the robustness of our conclusions.

### 3.6 Publication bias

As the number of included studies was too insufficient with no more than 10, the assessment of publication bias may be imprecise and meaningless. Therefore, we did not assess overall publication bias.

### 3.7 GRADE rating

GRADEpro was used to assess these outcomes’ evidence certainty. The certainty of LVEDD was moderate, while the certainty of LVEF, BNP/NT-proBNP, 6MWTD, and LVESV were low. Additionally, the certainty of evidence was very low for LVEDV. The GRADE assessment of the evidence for outcomes are shown in Table 2.

**TABLE 2:** Quality of evidence.

Quality assessment							No of patients		Effect		Quality	Importance
No of studies	Design	Risk of bias	Inconsistency	Indirectness	Imprecision	Other considerations	LVEF	Control	Relative (95% CI)	Absolute		
LVEF (Better indicated by lower values)												
8	Randomised trials	Serious	Serious	No serious indirectness	No serious imprecision	None	442	446	-	MD 6.23 higher (4.35 to 8.12 higher)	⊕⊕ΟΟ LOW	CRITICAL
BNP/NT-pro-BNP (Better indicated by lower values)												
5	Randomised trials	Serious	Serious	No serious indirectness	No serious imprecision	None	303	309	-	MD 187.94 lower (267.2 to 108.67 lower)	⊕⊕ΟΟ LOW	CRITICAL
6MWTD (Better indicated by lower values)												
3	Randomised trials	Serious	No serious inconsistency	No serious indirectness	Serious	None	137	135	-	MD 25.26 higher (8.23 to 42.3 higher)	⊕⊕ΟΟ LOW	CRITICAL
LVEDV (Better indicated by lower values)												
3	Randomised trials	Serious	Serious	No serious indirectness	Serious	None	174	172	-	MD 22.83 lower (42.79 to 2.87 lower)	⊕ΟΟΟ VERY LOW	CRITICAL
LVEDD (Better indicated by lower values)												
3	Randomised trials	Serious	No serious inconsistency	No serious indirectness	No serious imprecision	None	195	195	-	MD 4.76 lower (5.77 to 3.74 lower)	⊕⊕⊕Ο MODERATE	CRITICAL
LVESV (Better indicated by lower values)												
2	Randomised trials	Serious	No serious inconsistency	No serious indirectness	Serious	None	99	97	-	MD 11.86 lower (19.89 to 3.83 lower)	⊕⊕ΟΟ LOW	CRITICAL

## 4 Discussion

PQ is a perennial herbaceous plant belonging to the Araliaceae family. PQS are its primary active constituents, and research suggests that PQS may be beneficial for cardiovascular diseases, with its protective effects mainly attributed to its antioxidant properties (Lu et al., 2023). Furthermore, previous studies have indicated that PQS can reduce the adverse effects of cardiac damage induced by anti-cancer drug (cisplatin) (Xing et al., 2019) and also provide protection against cisplatin-induced acute kidney injury (Ma et al., 2017). The stems and leaves of American ginseng can be harvested from September to October each year, and PQS extracted from these stems and leaves has the advantage of lower cost (Zhang et al., 2022). Currently, in China, the only Chinese Proprietary Medicine approved for marketing containing PQS is Xinyue Capsules. Therefore, we conducted a comprehensive evaluation of the efficacy and safety of PQS preparations for HF using systematic review and meta-analysis methods.

### 4.1 Summary of evidence

This study identified a total of nine RCTs, encompassing 952 HF patients, to evaluate the efficacy and safety of PQS for HF. Significant differences in clinical efficacy were observed across all PQS groups, with improvements noted in patients’ cardiac function. Meta-analysis results from eight RCTs indicated that PQS for HF led to enhanced cardiac function and improved clinical symptoms and signs, with statistically significant differences (P < 0.05).

The systematic review showed that compared to conventional therapy alone, the combination of PQS and conventional treatment significantly improved LVEF, 6MWTD and reduced BNP/NT-pro-BNP, LVEDV, LVEDD, and LVESV with HF patients. Utilizing the GRADEpro, we assessed the quality of evidence for these outcomes, with four outcome measures rated as low, one as moderate, and another as very low. The findings of this review suggested that PQS combined with conventional therapy not only improves cardiac function and reduces cardiac load but also enhances exercise tolerance and elevates the quality of life among patients, demonstrating superior efficacy over conventional therapy. However, heterogeneity was detected in the examination of LVEF, BNP/NT-pro-BNP, LVEDV, and LVEDD outcomes. Consequently, we conducted subgroup analyses solely on LVEF, focusing on studies with larger sample sizes, based on treatment duration. The subgroup analysis revealed non-significant heterogeneity for treatments lasting less than 12 weeks but significant heterogeneity within subgroups exceeding 12 weeks. Additionally, potential sources of heterogeneity, such as small sample sizes, variations in age, gender, duration of disease, and the presence of comorbidities, could introduce bias and compromise the reliability of the results.

Only one trial compared the incidence rates of adverse reactions between two groups. Although the incidence rate of adverse reactions in the PQS treatment group was slightly lower than that in the control group, the difference was not statistically significant. The study also provided specific clinical manifestations of adverse events, with dry cough, gastrointestinal reactions, hypokalemia, and hypotension being common adverse reactions. However, since both the intervention group and the control group received conventional drug therapy, which included diuretics and RASI (ARB/ACEI), these drugs themselves may lead to similar adverse reactions such as hypotension and hypokalemia. Therefore, it is difficult to determine whether these adverse events were solely caused by PQS or side effects of other conventional medications. Additionally, three trials did not observe any adverse reactions or side effects. Hence, the safety of PQS for HF is currently difficult to ascertain.

### 4.2 Limitations

Our study results consistently demonstrated that adding PQS preparations to conventional anti-HF medications significantly improved clinical efficacy, enhanced cardiac function, and thus improved the quality of life of patients, with no apparent adverse effects. However, this review has the following several limitations. Firstly, due to the inclusion of a limited number of trials, and the generally poor methodological quality of most, along with heterogeneity in some outcome measures, the persuasive power of the meta-analysis results may be somewhat limited. Secondly, all intervention groups received PQS in combination with conventional medication. Thus, it remains unclear whether the efficacy of PQS was influenced by interactions with conventional drugs. Thirdly, although all included studies were RCTs, the GRADE assessment revealed that the quality of evidence for some outcomes was low or very low. This was primarily due to the following reasons: due to some studies (Han et al., 2011; Guo, 2012; Shi, 2014; Wang, 2018) lacked specific descriptions regarding the generation of random sequences, concealment of randomization, blinding procedures, etc. These problems increase the risk of bias and reduce the reliability of the study results. In addition, significant heterogeneity was observed in LVEF, BNP/NT-pro-BNP, and LVEDV outcomes and could not be explained by subgroup analysis. Thus, there was significant inconsistency in LVEF, BNP/NT-pro-BNP, and LVEDV outcomes. Moreover, the large SD of one study (Han et al., 2011) included in the 6MWTD, LVEDV, and LVESV analyses, as well as the small number of studies and small sample sizes used for the analyses, resulted in wide MD 95% CI. These issues contributed to the imprecision of the studies. Fourthly, all included RCTs were conducted in China with Chinese participants, making it difficult to ascertain the influence of ethnicity and region, which may limit the generalizability of the results. Fifthly, because the original RCT studies included did not provide detailed data on reported efficacy stratified by age, gender, and HF severity, it was not possible to explore differences in response to PQS in different patient groups. Furthermore, there was a lack of descriptions of adverse reactions of PQS. Hence, conclusions regarding adverse reactions remain inconclusive, necessitating further observation of its safety profile.

### 4.3 Suggestions for future clinical research

While our systematic review and meta-analysis results showed that PQS is safe and effective in the treatment of HF, there was a high degree of heterogeneity in some outcomes and methodological limitations inherent in the RCTs included. Therefore, its safety and efficacy should be approached with caution. In the future, we recommend conducting high-quality RCTs with large sample sizes, multicenter involvement, double-blinding, and placebo controls to confirm the efficacy and safety of PQS for HF. Additionally, it is advisable to register trial protocols before conducting clinical research and to rigorously monitor adverse reactions during the study. Researchers should also not overlook assessing the interaction effects of PQS when used in conjunction with conventional HF medications, whether beneficial or adverse, in order to make a reasonable assessment of the safety of the drug. Furthermore, since the study participants were all Chinese, international research could be pursued in the future. In addition, to address the limitations in the GRADE assessment and to improve the quality of evidence from future studies, we suggest that future clinical research should prioritize rigorous study designs, including adequate randomization, blinding, and allocation concealment, to minimize the risk of bias. At the same time, increased sample sizes are needed to improve the precision of effect estimates and enhance the statistical power of analyses. For future research, we should also prioritize the collection and reporting of subgroup data to identify which populations may benefit most from PQS therapy. Lastly, we observed that all included trials lacked long-term follow-up and did not report long-term benefits. Therefore, future research should evaluate the long-term effects of PQS preparations for HF treatment, such as HF progression, HF readmission rates, 3-year survival rates, 5-year survival rates, and mortality events. We will continue to monitor this study closely.

## 5 Conclusion

The evidence provided by this systematic review suggests that adjunctive PQS therapy for HF can improve clinical efficacy and hold potential advantages in improving cardiac function and increasing exercise tolerance. However, given the limitations inherent in this review and the results of the GRADE assessment, the conclusions of this study should be interpreted cautiously. Therefore, in clinical practice, it is recommended that physicians tailor treatment strategies according to the specific circumstances of individual patients. Furthermore, it is advised to conduct further high-quality RCTs with large sample sizes, multicenter involvement, double-blinding, placebo controls, and long-term follow-up to provide robust clinical evidence support for the efficacy and safety of PQS for HF.

## Data Availability

The original contributions presented in the study are included in the article/Supplementary Material, further inquiries can be directed to the corresponding author.
