# Supplementary figures and images for: The efficacy and safety of panax quinquefolius saponin for heart failure: a systematic review and meta-analysis
Source: Front Pharmacol. 2025 Feb 28;16:1463609. doi: 10.3389/fphar.2025.1463609 (PMC11906658; doi:10.3389/fphar.2025.1463609)

Panax quinquefolius  
saponin

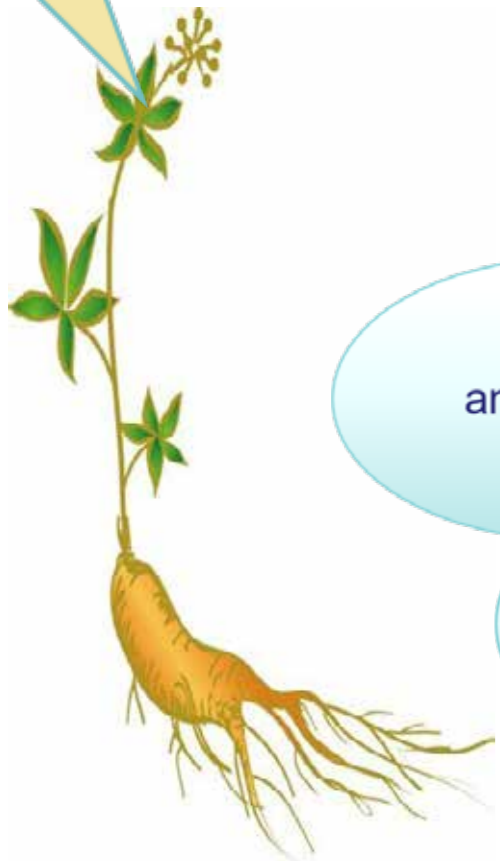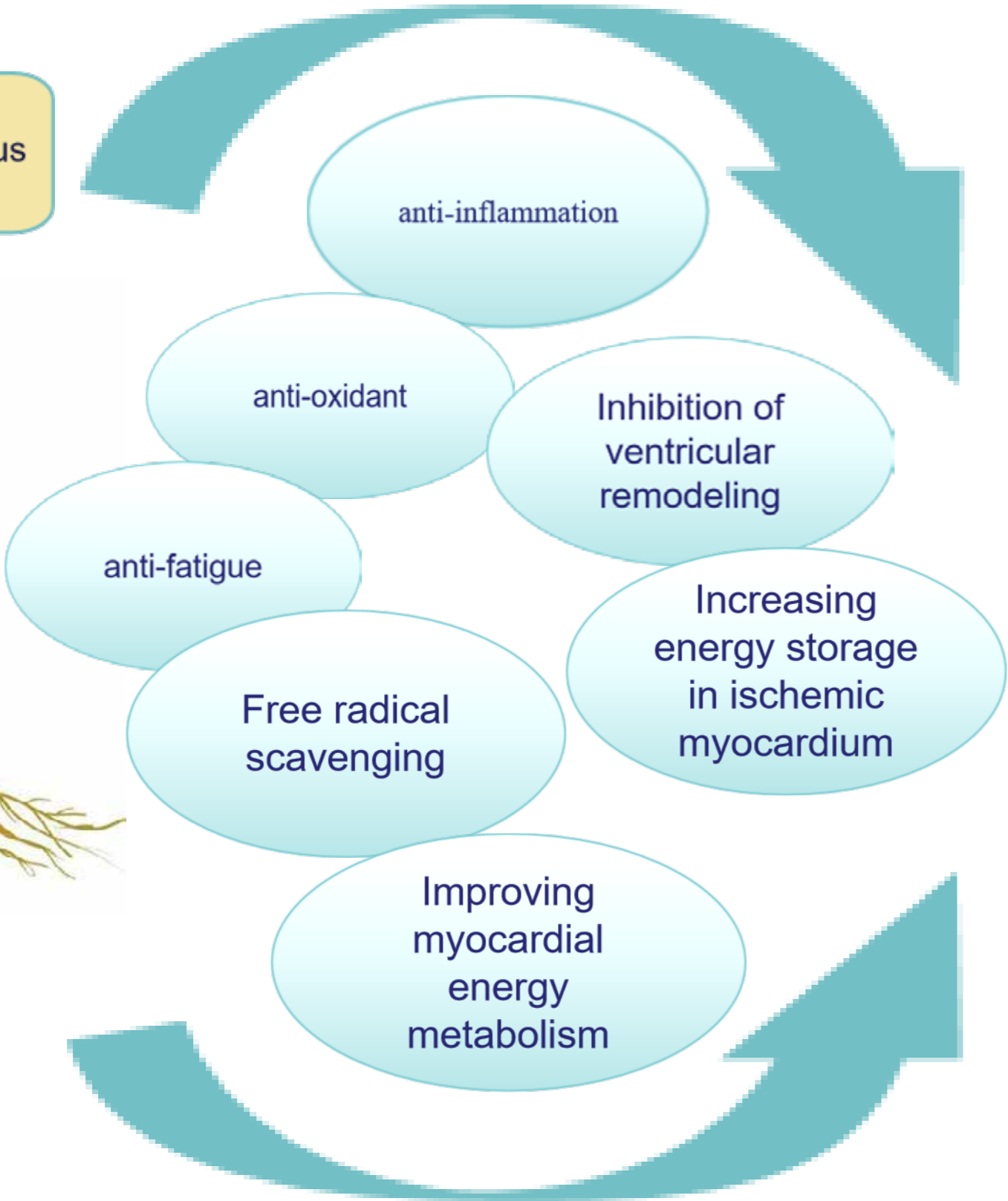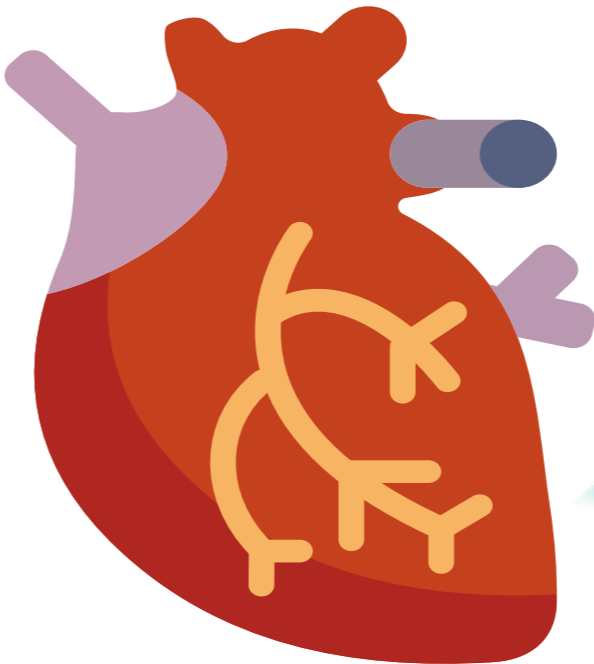

Heart failure

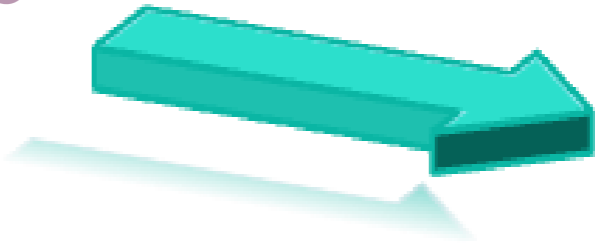

LVEF  
6MWTD

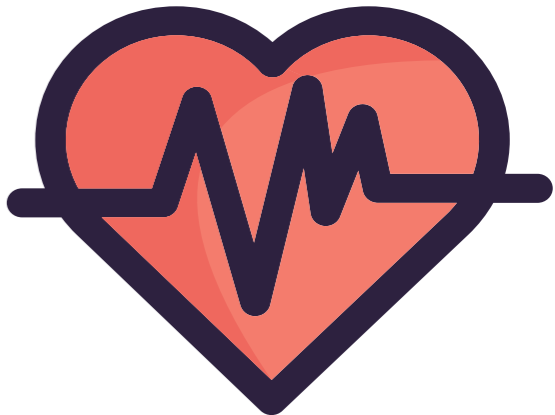

BNP/ NT-pro-BNP  
LVEDV  
LVEDD  
LVESV

Supplement: Supplementary file 1 [file DataSheet1.pdf]
